# Endophytes: Colonization, Behaviour, and Their Role in Defense Mechanism

**DOI:** 10.1155/2020/6927219

**Published:** 2020-07-30

**Authors:** Anteneh Ademe Mengistu

**Affiliations:** Adet Agricultural Research Center, P.O. Box 08, Bahir Dar, Ethiopia

## Abstract

Biotic and abiotic factors cause an enormous amount of yield and economical loss. However, endophytes can play a significant role in enhancing the tolerance of plants. Endophytes systematically colonize different parts of the host, but plants use a variety of defense mechanisms towards microbial infection. However, they have to survive the oxidative environments, and endophytes like *Enterobacter* sp. encode superoxide dismutases, catalases, and hydroperoxide reductases to cope up the oxidative stress during colonization. On the contrary, others produce subtilomycin which binds with flagella to affect flg22-induced plant defense. The behavior of endophytes can be affected by different genes in hydrolase activity when they come into contact with the host plant. The lifestyle of endophytes is influenced by environmental factors, the host, and microbial genotypes, as well as an imbalance in nutrient exchange between the microbe and the host. For instance, induction of PiAMT1 in root endophyte *Piriformospora indica* indicates depletion of nitrogen which plays as a triggering factor for activation of the saprotrophic program. Microbes enhance disease resistance through induced systemic resistance (ISR), and *Bacillus cereus* triggers ISR against *Botrytis cinerea* through an accumulation of the PR1 protein and activates MAPK signaling and WRKY53 gene expression by the JA/ET signaling pathway. Similarly, *Trichoderma arundinaceum* produces trichodiene that affects *Botrytis cinerea* through induction of defense-related genes encoding salicylic acid (SA) and jasmonate (JA). Overall, endophytes can play a vital role in disease management.

## 1. Introduction

Crops are colonized by complex microbial communities [1], and some of them are detrimental and cause diseases, whereas others promote plant growth and enhance nutrient acquisition and tolerance to biotic and abiotic stresses via a multitude of mechanisms [2]. The fungi or bacteria which grow inside the plant tissue without causing any harm to the host are termed as endophytes. They associate with the majority of plant species found in natural and managed ecosystems. Endophytes are considered as important plant partners that play an important role in improving stress tolerance of the host compared to those that lack such symbiosis [3, 4]. Most endophytes are found without any known effect, but numerous bacteria and fungi establish a mutualistic or pathogenic association with the host plant. Mostly, the outcome of interactions relies on the environmental factors, the genotype of both the host and the interacting microorganism [2].

Plants could sense microbes via the perception of microbial-/pathogen-associated molecular patterns (MAMPs/PAMPs) by pattern-recognition receptors (PRRs). PRRs are classes of cell surface recognition proteins involved in initial signaling that trigger the ﬁrst layer of plant innate immunity. Flagellin protein (flg22) and elongation factor Tu (EU-Tu) are the two most well-characterized MAMPs/PAMPs [5]. In general, during the establishment of symbiosis, most of the pathways targeted by miRNAs for plant defense systems are turned off that would otherwise have obstructed proliferation of endophytes [6].

Endophytes are found in all plant species regardless of their place of origin. The ability to enter and thrive in the host tissues makes them unique, showing multidimensional interactions within the host plant. Several host activities are known to be inﬂuenced by the presence of endophytes. They can promote plant growth, elicit defense response against pathogen attack, and can act as remediators of abiotic stresses [7]. Overall, fossil records of endophytes date back to more than 400 million years, implicating these microorganisms in host plant adaptation to habitat transitions [4].

## 2. Colonization Mechanism

Microbes, whether they are beneficial or plant pathogens, have similar potentials like rhizosphere competence, motility to reach the host plant, mechanisms for entrance and spreading inside the plant, and the ability to overcome plant immunity [8, 9]. Successful colonization by endophytes is affected by different factors including the plant tissue type, the plant genotype, the microbial taxon and strain type, and biotic and abiotic environmental conditions [1]. Similarly, growth medium, plant age and species, inoculum density, and fungal species, as well as the rate of conidia application, affect endophytic colonization [10]. Bamisile et al. [11] reported the influence of seedling age on *B. bassiana* and *M. anisopliae* successful colonization in the citrus plant. From their point of entry, microbes may systemically colonize plants from roots to shoots, shoots to flowers or fruits, and/or from flowers to fruits and seeds, and they may also cause localized colonization inside/outside plant organs [12]. Colonization of olive (*Olea europaea* L.) through root hair with *Pseudomonas fluorescens* PICF7 and *P. putida* PICP2 enables the plant to withstand soil-borne fungal pathogen *Verticillium dahliae* Kleb [13]. On the contrary, in berries, some *Firmicutes* and *Bacillus* spp. are reported to colonize cell walls of the seed endosperm and consistently found inside flower ovules as well as in the pulp and inside seeds [14]. Thereby, the colonization of endophytes is organ- and tissue-specific due to selective pressure. This tissue specificity in colonization leads to tissue-specific protection from diseases [15, 16].

Plants use a variety of defense mechanisms against microbial infection, and the response of the host plant drastically differs to the colonization of endophytes and a pathogen. Prior to colonization, microbes have to survive the oxidative environments within the host plant. For instance, *Enterobacter* sp. encode superoxide dismutase, catalases, and hydroperoxide reductases to cope up the oxidative stress during colonization of poplar (*Populus trichocarpa*×*deltoides* cv. H11-11) [17]. Chen et al. [18] reported rice blight *Xanthomonas oryzae* pv. oryzae PXO99 induced a much stronger defense reaction than the endophyte *Azoarcus olearius* in rice plants. Surprisingly, differentially expressed genes (DEGs) related to the jasmonate (JA) signaling pathway are constantly activated by beneficial endophytes in contrast to the salicylate (SA) pathway which is activated only in rice roots of infected plants by the pathogen indicating that JA is involved in controlling the *Azoarcus* endophyte density in roots. In *Arabidopsis thaliana*, endophyte bacterium *Bacillus subtilis* BSn5 produces subtilomycin which affects flg22-induced plant defense by binding with flagellin and ultimately enhances its ability to colonize plant endosphere [19].

Endophytic strain *Serratia plymuthica* G3 and QS genes control important colonization-related traits such as swimming motility and biofilm formation. Likewise, genes for superoxide dismutases, putative catalases, peroxidases, and reductases are used by diazotrophic *Klebsiella pneumoniae* (Kp) 342 to protect its cells against plant ROS [20]. On the contrary, in sugarcanes, the shr5 gene is differentially expressed to the colonization of beneficial and nonbeneficial microbes. This gene encodes a protein involved in plant signal transduction during the establishment of plant-endophyte interactions. Downregulation of shr5 occurred exclusively when inoculated with beneficial bacteria like *Gluconacetobacter diazotrophicus* [5]. According to Kandel et al. [21], during the early stages of rice root colonization, an endophytic bacterium *Gluconacetobacter diazotrophicus* also expressed ROS-deactivating genes such as superoxide dismutase (SOD) and glutathione reductase (GR) in greater amounts. Likewise, endoglucanase plays a major role in endophytic colonization. An *eglA* mutant failed to efficiently invade the plant cells and systematically colonize the plant, in contrast to the wild-type strain. *Azoarcus* sp. endoglucanase is an important determinant for successful endophytic colonization of rice roots [20, 22].

## 3. Endophytic Behavior

Most plant pathogens carry genes encoding plant cell wall-degrading enzymes. However, nonphytopathogens may possess glycoside hydrolase other than cellulase/hemicellulase (or cell wall degradation hydrolases). The presence of this enzyme in numerous endophytes is consistent with its possible role in the diversity of sugar utilization that might be a useful component of a competent endophyte [23]. According to Taghavi et al. [17], the genome of *Enterobacter* sp. does not encode proteins involved in cellulose degradation, which is consistent with its nonpathogenic behavior, during the interaction of the endophyte and the poplar tree. The endophytic behavior can be affected by different genes that are found to be conserved including various transcriptional regulators. For example, the presence of the LrgB family protein is mainly involved in controlling hydrolase activity whose most likely function occurs when endophytes come into contact with plant hosts at the time of plant infection [23].

**Figure 1:**
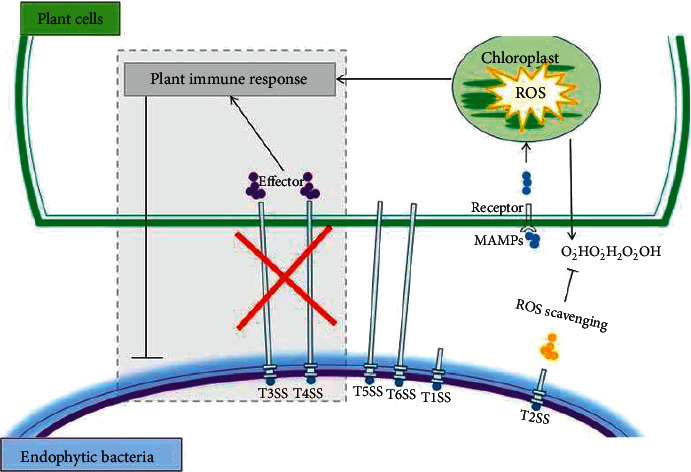
Plant's reaction as a result of the gene encoding secretion systems in endophytes and plant pathogenic bacteria, adopted from Liu et al. [25].

Protein secretion plays a major role in defining plant-microbe interactions. The transport of effector proteins plays an important role in the parasitic lifestyle of bacteria by suppressing host defense, whereas the effector-triggered immune responses are stimulated by the host whenever it recognizes the effector proteins. Particularly important in this context are the T3SSs and T4SS [24] (Figure 1). On the contrary, genes for T3SSs are largely missing or incomplete in genomes of mutualistic endophytes. They can be considered as disarmed pathogens that lost their functional T3SSs, and they evolved to an endophytic lifestyle. For instance, T3SS mutants of *Salmonella enterica* showed increased endophytic colonization in *Medicago truncatula*. Generally, Type I and Type II secretion systems are present in several bacterial endophytes [2, 9], but Type III and Type IV secretion systems are mainly present in pathogenic bacteria and are mostly absent in endophytes [23, 26].

The plant receptor FLS2 recognizes flagellin of bacteria and initiates plant defense. It has been reported that the recognition of *P. syringae* flagellin in *Arabidopsis* and *Nicotiana benthamiana* triggered stomata closure [27] and activation of MAP kinase [2]. This leads to transcriptional induction of pathogen-responsive genes, production of reactive oxygen species, and deposition of callose to reinforce the cell wall and prevent microbial growth at infection sites. However, flagellin of the mutualist endophyte *Paraburkholderia phytofirmans* PsJN triggered a weak and transient defense reaction with an oxidative burst but to a lower extent compared to pathogenic interactions [2, 28, 29]. In line with this, the downregulation of flagella biosynthesis and upregulation of functions related to flagellar motor rotation assist endophytes to hide their flagellin PAMPs and move faster in plant environment, whereas downregulation of elongation factor EF-Tu enabled the colonization of rice by endophytic bacteria [30].

LPSs are also known to induce different host responses for pathogens and nonpathogen endophytes. LPSs from the plant beneficial strain of *P*. *phytofirmans* PsJN can downregulate defense genes, such as defense-like PR1, superoxide dismutase, and the COP9 signalosome complex in potato leaves which indicates that plants can identify LPSs derived from nonpathogenic endophytes [2]. Overall, genes putatively involved in antibiotic resistance (evgS and evgA), redox response (regB and regA), nitrogen ﬁxation and metabolism (ntrY and ntrX), and cell fate control (pleC and pleD) are found more prominently among endophytes than among phytopathogens [1].

## 4. Switch among Lifestyle in Fungi

Fungi use different survival strategies and lifestyle patterns after entering into the plant system to associate intimately with the plants. Endophytes profit from host plants by receiving organic nutrients, protective shed, and guaranteed transmission to the next host generation; on the contrary, infected host plants are more vital, stress-resistant, and toxic to herbivores, nematodes, and pathogens [3]. The fungal endophytes have a broad host range, and they may choose one of the many strategies for entering into the host internal system such as the production of toxic metabolites, modification of plant elicitors, and suppressing the plant immune system [31]. Host preference is an important parameter for both parasitic and symbiotic plant-fungal interactions [32], and it originates from the close adaptation between the host plant and its fungal partner through cohabitation and coevolution, which finally leads to stronger partnership and is permanently imprinted in the genetic constitution of both partners.

Host and microbial genotypes are the most important factors responsible for the expression of a particular lifestyle. The interaction can be considered as a flexible interaction, whose directionality, to some extent, is determined by slight differences in the fungal gene expression in response to the host and also by host recognition and response to the fungus. Several studies examining the relation between the host genotype and the symbiotic lifestyle expression demonstrated that individual isolates of some fungal species could express either parasitic or mutualistic lifestyles depending on their host genotype [33]. The genetic and biochemical base of a fungal lifestyle change from endophytic to parasitic is characterized by an imbalance in the nutrient exchange between the plant and the fungus. According to Rai and Agarkar [3], UV mutagenesis of a virulent isolate (CmL2.5) of *C*. *magna* leads to the enhancement of host plant fitness to disease and drought. Similarly, asymptomatic endophyte *Diplodia mutila* switches its lifestyle to pathogenic. Alvarez-Loayza et al. [34] reported that high light triggers endophyte pathogenicity, while low light supports endosymbiotic development. The pathogenicity under high light resulted from light-induced production of H_2_O_2_ by the fungus, triggering hypersensitivity, cell death, and tissue necrosis. Their study demonstrated that endophytes respond to abiotic factors to influence plant-fungal interactions in natural ecosystems, and the light was identified as the influencing factor. In general, changing of lifestyle from endophytic to pathogenic or vice-versa when colonizing its host might be due to the disruption of a balanced communication with its host factor [35].

The root endophyte *Piriformospora indica* requires the provision of an adequate source of nitrogen to induce low expression of the *P. indica* high-affinity ammonium transporter during host colonization [36]. On the contrary, the induction of PiAMT1 indicates the depletion of nitrogen which plays as a triggering factor for the in planta expression of fungal genes that encode hydrolytic enzymes for the activation of the saprotrophic program. Silencing of PiAMT1 results in reduced expression of fungal xylanase and host's defense response. Hence, the expression and a signaling function of PiAmt1 are needed for the switch of *P. indica's* lifestyle to saprotrophy [37]. The disruption of communication between *Pinus sylvestris* and *Neurospora crassa* plays a role in changing the lifestyle from endophytic to pathogenic [35]. Disruption of components of the Nox complex (NoxA, NoxR, and RacA), or stress-activated MAP kinase (SakA), leads to a breakdown in this finely balanced association, resulting in pathogenic infection instead of mutualism. In the sakA mutant association, a dramatic upregulation of fungal hydrolases and transporters was observed, changes consistent with a switch from restricted symbiotic to proliferative pathogenic growth [38]. Sometimes, a microbe varies its association with different hosts. Temporal induction of genes, carbohydrate-active enzymes (CAZymes), and necrosis-inducing effectors plays a vital role in infection and colonization of hosts. *Fusarium virguliforme* effectors and CAZymes are expressed in temporal distinct waves immediately after infection in the soybean-infected root compared to maize. On top of that, the upregulation of Zn(II)-Cys6 genes during early soybean colonization might play a role in the enhancement of pathogenicity of *F. virguliforme* on soybean [39].

## 5. Induction of Plant Disease Resistance

Endophyte microbiomes are known to significantly influence host performance especially under stressed conditions [40, 41] and mediate functioning of the plant microecosystem by critically altering the responses of the plant to environmental changes [42]. Endophytes have to compete with plant cells for Fe supply, and therefore, siderophore production is highly important for endophytic growth through increasing availability of minerals in addition to iron chelation and also involved in suppression of pathogens by stimulating the biosynthesis of other antimicrobial compounds [43–45]. Extensive multiplication and colonization of plant tissues by endophytes result in a “barrier effect,” where the existing endophytes compete with the pathogenic microorganisms and prevent them from taking hold. Similarly, endophytes play an imperative role to maintain the health of plants, through antibiosis or induced systemic resistance, as they can protect or prepare the plant against biotic stresses and help in enhancing growth and yields [46].

**Figure 2:**
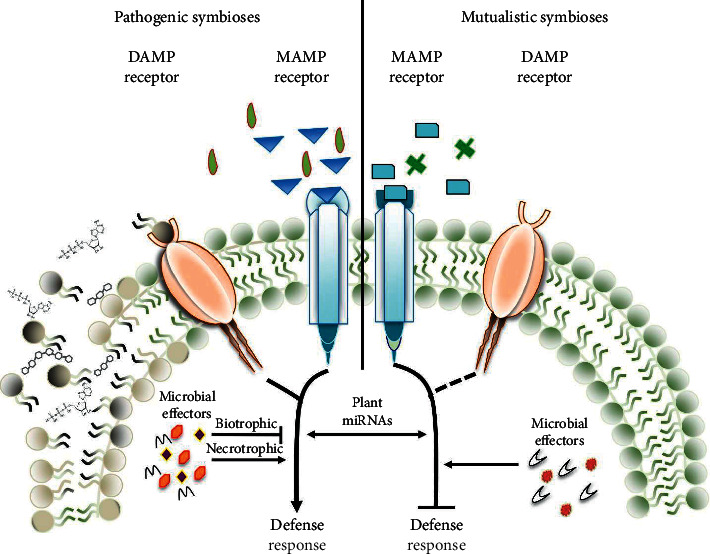
Recognition between mutualistic and pathogenic interaction of the host during induction of the defense response, adopted from Plett and Martin [6].

Microbes enhance disease resistance through the mechanism of induced systematic resistance (ISR) and systemic acquired resistance (SAR) [47]. Microbe- or pathogen-associated molecular patterns (MAMPs/PAMPs) are essential structures that are conserved and necessary for microbial survival, but plants have evolved multiple families of receptor proteins to recognize them and induce the plant immune system [6] (Figure 2). Pattern-recognition receptors (PRRs) have evolved to recognize common microbial compounds, such as bacterial flagellin or fungal chitin, called pathogen- or microbe-associated molecular patterns (PAMPs or MAMPs). Pattern recognition is translated into a first line of defense called PAMP-triggered immunity (PTI), which keeps the most potential invaders under control [2].

Tomato and cotton seed treatment with *Beauveria bassiana* induced protection against *Pythium myriotylum* and *Rhizoctonia solani* [10]. Similarly, *Beauveria bassiana* is known to induce *Citrus limon* plant resistance to an insect pest *Diaphorina citri* [48]. Endophytic fungus *Lecanicillium longisporum* suppresses powdery mildew and aphids in cucumber plants [10]. The endophyte *Bacillus cereus* triggers ISR against *Botrytis cinerea* on *Arabidopsis thaliana* through an enhanced accumulation of the PR1 protein expression on time, hydrogen peroxide accumulation, and callose deposition. Mitogen-activated protein kinase (MAPK) cascades play a crucial role in the biotic as well as abiotic stress response through decoding external stimuli and signal transduction [49]. Endophyte activates MAPK signaling and the WRKY53 gene expression, both of which are involved in the pathogen-associated molecular pattern- (PAMP-) triggered immunity (PTI) by the JA/ET signaling pathway in an NPR1-dependent manner [50]. The bacterial endophyte *Azospirillum* sp. also induces systemic disease resistance in rice against rice blast, and ET signaling is required for endophyte-mediated induced systemic resistance (ISR) in rice [51]. Overall, the combination of jasmonic acid (JA) and ethylene (ET) signaling activates resistance against necrotrophic pathogens, whereas salicylic acid (SA) signaling triggers resistance against biotrophic and hemibiotrophic pathogens [52].

Endophytes induce several cell wall modifications, such as deposition of callose, pectin, cellulose, and phenolic compounds, leading to the formation of a structural barrier at the site of a potential attack by phytopathogens. Similarly, they induce defense-related proteins such as peroxidases, chitinases, and *β*-1,3-glucanases [20]. *Trichoderma arundinaceum* produces VOCs like trichodiene that affects *Botrytis cinerea* through induction of the expression of tomato plant defense-related genes encoding salicylic acid (SA) and jasmonate (JA) [53]. Barley inoculated with oxo-C14-homoserine lactone (AHL) producing *Ensifer meliloti* enhances the resistance against *Puccinia hordei* [54]. The genome analysis of the endophytic biocontrol strain of *Pseudomonas chlororaphis* subsp. *aurantiaca* PB-St2 revealed the presence of acyl-homoserine lactone (AHL) biosynthesis genes *phzI*, *csaI*, and *aurI* involved in clear AHL production which might have a role for biocontrol activity [55]. Similarly, endophytic *Pseudomonas putida* modified with an antifungal phz gene obtained from *Pseudomonas fluorescens* plays a major role in the reduction of the fungal population on soils in wheat field [56].

Lipid transfer protein (LTP) plays a role in plant responses to biotic and abiotic stress. LTP1 binds jasmonic acid, and together, they compete with a stronger affinity for the elicitin binding site and are capable of inducing resistance at a distance from the point of application. Expression of CaLTP-N, encoding an LTP-like protein, reduced disease development, suggesting LTP is a functional component of resistance induced by *Trichoderma* species to *Phytophthora* infection in hot pepper [57]. The endophyte fungi *Penicillium citrinum* LWL4 and *Aspergillus terreus* LWL5 reduced fungal infection caused by *Alternaria alternata* in the sunflower plant by regulating oxidative stress responses by activating glutathione and polyphenol oxidase and downregulating catalase and peroxidase. Similarly, the amino acid content was higher on leaves inoculated with endophytes which suggests such change delays cell death and disturbs fungal progression in the plant tissue [58].

Seed-borne endophytic microbe *Bacillus amyloliquefaciens* RWL-1 induces disease resistance against pathogenic *Fusarium oxysporum f*. sp. *lycopersici* in the tomato plant through activation of amino acid biosyntheses like aspartic acid, glutamic acid, serine (Ser), and proline (Pro). They are important in the induction of plant defense during pathogenesis. Pro plays a role in strengthening of the cell wall during pathogen attack [59]. The level of defense-related oxidative enzymes like phenyl ammonia lyase (PAL), Polyphenol oxidase (PPO), and peroxidase (PO) was higher on tomato plants treated with bacterial endophytes which resulted in induced systemic resistance against *Fusarium* wilt of tomatoes [60, 61].

## 6. Conclusion

Successful establishment of endophytes within the host is affected by the tissue type, the genotype of the host, and microbe, as well as the environmental conditions. Crops colonized by endophytes have a high tendency to stress tolerance than those that lack such symbiosis. Most pathways targeted by miRNAs for plant defense are turned off during the establishment of symbiosis. Similarly, genes involved in anabolic pathways are more diverse and abundant among endophytes in contrast to phytopathogens. The endophytic behavior can be affected by different genes that are found to be conserved including various transcriptional regulators. Sometimes, endophytes can downregulate flagella biosynthesis and upregulate functions related to flagellar motor rotation to hide their flagellin PAMPs and move faster within plants during colonization. Endophytes use different survival strategies and lifestyle patterns after entering into the plant. They will change their lifestyle into pathogenic whenever an imbalance occurred during the host-microbe interaction.

Endophytes are known to influence host performance under stress conditions by altering the response of the plant to environmental change. They can act as a barrier as well as compute with pathogenic microorganisms and prevent them from taking hold. Similarly, through antibiosis or induced systemic resistance, they can maintain the health of the plant and assist in enhancing growth and yields. Jasmonic acid (JA) and ethylene (ET) as well as salicylic acid (SA) signaling is required for endophyte-mediated induced resistance. On the contrary, endophytes can induce disease resistance through the activation of amino acid biosynthesis. Overall, endophytes are regarded as extremely important plant partners with the potential to minimize the yield loss through the provision of improved stress tolerance to the host in an environmentally friendly manner and thereby enhance the productivity of agriculture.

## Data Availability

The data used to support the findings of this study are available from the corresponding author upon request.
